# ConfeitoGUI: A toolkit for size-sensitive community detection from a correlation network

**DOI:** 10.1371/journal.pone.0206075

**Published:** 2018-10-23

**Authors:** Yoshiyuki Ogata, Kazuto Mannen, Yasuto Kotani, Naohiro Kimura, Nozomu Sakurai, Daisuke Shibata, Hideyuki Suzuki

**Affiliations:** 1 Graduate School of Life and Environmental Sciences, Osaka Prefecture University, Osaka, Japan; 2 Department of Research and Development, Kazusa DNA Research Institute, Chiba, Japan; Southwest University, CHINA

## Abstract

Analysis of the large amounts of data accumulated in public databanks can facilitate a more comprehensive understanding of molecular biological processes. Community detection from molecular biological data is paramount in characterizing evolutionary and functional traits of organisms based on gene homology and co-expression, respectively. Although there are common tools to detect local communities from a large network, no toolkit exists for detecting communities that include an element of interest based on size sensitivity, i.e., functionality to obtain local communities with preferred sizes. Herein, we present the ConfeitoGUI toolkit for detecting local communities from a correlation network involving size sensitivity. We compared the toolkit with other common tools for detection in reconstructing communities of microarray experiments of mice. In the results, ConfeitoGUI was observed to be preferable for detecting communities whose sizes are similar to those of original communities compared to other common tools. By changing simple parameters representing sizes for the toolkit, a user can obtain local communities with preferred sizes, which is beneficial for further analysis of members belonging to the communities.

## Introduction

In the era of big data, biologists encounter challenges in handling, processing, and moving such data obtained via high-throughput technologies [1]. By late last century, network graphs were used to visualize complex systems based on large social datasets. Clauset et al. [2] suggested that approaches using network graphs are useful not only for social science, but also for biochemistry and molecular biology. This was followed by various approaches to detect local communities from large networks such as those by Newman et al. [3] and Blondel et al. [4]. In these approaches, the modularity index is used to show the reasonability of local communities divided by their algorithms. On the other hand, these approaches provide no attributes for individual elements in the network. Therefore, elements around the borders of multiple local communities can be misclassified because information for appropriate classification is insufficient. Using a network model of Zachary’s karate club, Newman et al. [3] divided the club members into two communities according to their method based on the shortest-path betweenness, and, consequently, only one member was misclassified in comparison with the club’s actual division. After that, Newman [5] proposed an eigenvector-based algorithm, which can successfully classify the network model.

In our previous research [6], we constructed the Confeito algorithm to detect local communities, which provides attributes of each element in a network, and thus can determine a classification for each element. Network density is a common index to characterize a local community or the whole network. The index can be separated into values for individual elements, i.e., providing edges for the community members rather than simply the number of members. We devised an index as a dual measure of network density for a complete and exclusive (i.e., with no link to other local communities) network graph. Using network density and the dual index, the algorithm can quantify a local community in comparison with a perfect and exclusive network graph. By applying the algorithm to the network model of Zachary’s karate club, we successfully classified the network model, and showed that members located intermediate between two communities have their indecisive indices classified into either community. By elucidating the attributes of border-located elements, the Confeito algorithm is border-sensitive for local community detection. In other words, the algorithm is applicable to a network containing indistinct local communities such as those in the field of molecular biology.

The use of high-throughput analytical methodologies in molecular biology such as microarrays, high-throughput sequencing, and tandem mass spectrometry has led to the accumulation of large amounts of data in public databanks. The analysis of such data can provide new insights into molecular biological processes. Nucleic acid sequences are deposited in GenBank [7], maintained by the National Center for Biotechnology Information (NCBI), and in the European Molecular Biology Laboratory (EMBL/EBI) Nucleotide Sequence Database, whereas amino acid sequences are deposited in the Universal Protein Resource (UniProt) database [8]. In its Reference Sequence Database (RefSeq) [9], the NCBI maintains non-redundant sequences integrated from many nucleotide and amino acid sequences. The RefSeq database currently contains more than 60 million amino acid sequences, and that number continues to increase. Compositional and quantitative metabolite datasets are deposited in the MassBank [10] and MetaboLights [11] databanks, maintained by EMBL/EBI. When integrated into a reconstructed dataset based on elements such as genes or metabolites, these data can be applied to network analysis in systems biology research by calculating correlation coefficients for relationships between elements to construct correlation networks. Molecular elements that are found within a community share particular biological features. Within the community, molecular elements for which we have little or no knowledge regarding their function can be annotated based on known traits of other elements. The resulting networks can be used to annotate unknown elements by mapping such elements onto the network.

Communities that are identified from analyses of molecular biological datasets (e.g., those in plants [12–15]) are useful for characterizing functional and evolutionary traits, such as by co-expression of genes and co-accumulation of metabolites for functional traits and gene homology for evolutionary traits.

Network analysis of gene co-expression helps to identify novel functional traits for lesser-known genes. Hirai et al. [12] performed a network analysis using Arabidopsis microarray datasets to identify novel enzymes and transcription-activating factors that upregulate the glucosinolate biosynthesis pathway and constructed a correlation network based on the expression patterns of the identified genes. In their approach, gene-gene correlation coefficients were calculated based on their expression profiles, and then a correlation network between a local community including the genes of interest was depicted. The network included two novel transcription factors and several novel enzymes related to the glucosinolate pathway, and thus the authors were able to assign these genes to the pathway. Ogata et al. [13,14] constructed correlation networks composed of genes related to cellulose and monolignol biosynthesis in plants through a network analysis based on microarray datasets and the original Confeito algorithm [6].

The grouping of homologous genes provides information on evolutionary relationships based on quantitatively detecting common bases or amino acids. In general, matrixes for determining correlations between molecular biological elements consist of quantitative indices (e.g., ranging from -1 to 1 for the Pearson correlation coefficient). By varying the correlation index thresholds, communities of different size that represent different levels of functionality can be identified. On the basis of gene homology, Ogata and Suzuki [15] constructed a plant correlation network comprising 3,167 genes encoding cytochrome P450 (CYP) proteins. A correlation coefficient threshold of 0.5 in their network identified 217 CYP gene communities. Varying the threshold leads to the identification of different numbers of communities. The Cytochrome P450 Engineering Database (https://cyped.biocatnet.de/) [16] categorizes CYP genes into subfamilies (i.e., small groups), families (mid-sized groups), and superfamilies (large groups). Differences in the number of communities identified based on application of different thresholds can represent CYP gene families of different size. The assignment of a gene within multiple levels of a gene community can enhance our understanding and provide a broad perspective of the evolutionary and functional traits of the gene.

Local communities (referred to here as “network modules”) can be extracted from a network comprising elements and links between elements using a variety of approaches, as noted in previous reports [3,4,17–21]. Although these methods can extract network modules that include a focused element, it is difficult to adjust the size of the modules (i.e., the number of elements included); the modules are thus often much smaller or larger than expected. In the field of molecular biology, for example, elements such as genes or metabolites in a module that includes an element of a researcher’s interest should be further analyzed as candidates to verify the predictions made by the community assignment. When conducting such analyses, a reasonable number of elements is desired for actual experiments. A size-sensitive approach thus supports actual verification for such uses.

Consequently, we developed a standalone toolkit, ConfeitoGUI, to identify network modules within correlation networks in a size-sensitive manner by expanding the Confeito algorithm [6,22] and integrating vertex-vertex connections based on the algorithm. The preliminary program of the original Confeito algorithm, which was written as a Perl script, constructs a network module for an arbitrary element, and thus the network modules for all elements have redundancy in their memberships; i.e., a single element belongs to multiple network modules. ConfeitoGUI has a function to remove such redundancies in its backend process as well as implementation of its frontend process on a graphical user interface (GUI). The ConfeitoGUI tool allows the adjustment of network module size by manipulation of simple parameters representing sizes, and it can identify elements specifically related to the network modules even when they are weakly correlated. Focusing on a particular element, the user can modify the size of the module including the element.

## Materials and methods

### Confeito algorithm indices

According to Ogata et al. [6], the Confeito algorithm requires three basic indices to identify a local community from within a network graph (i.e., *ND*, *NS*, and *NF*, representing network density, network specificity, and network *F*-measure, respectively). Network density and network specificity are dual indices that consider completeness and exclusivity (or with no connections to other local communities). When imaging a network graph with complete intra-modular connections (perfect) and with no connections to other graphs (exclusive), network density and network specificity are “precision” and “recall” indices for the graph, respectively. Precision and recall are dual indices used for information retrieval and represent the ratio of true positive elements over all positive elements (i.e., for evaluating type I errors) and the ratio of true positive elements over all true elements (i.e., for evaluating type II errors), respectively. Because *F*-measure is a harmonic mean of the dual indices, the index can be used for evaluating type I and II errors. To evaluate the relationships between an element and a network module in which the element is included, vertex indices (*i*.*e*., *VD*_*i*_, *VS*_*i*_, and *VF*_*i*_ representing vertex density, vertex specificity, and vertex *F*-measure, respectively) were established using the present algorithm by resolving the former three indices into values based on individual elements:

$$ND=\frac{\sum e_{i}}{n∙(n-1)}$$ (1)

$$NS=\frac{\sum e_{i}}{\sum d}$$ (2)

$$NF=\frac{2}{\frac{1}{ND}+\frac{1}{NS}}=\frac{2∙\sum e_{i}}{n∙(n-1)+\sum d_{i}}$$ (3)

$${VD}_{i}=\frac{e_{i}}{n-1}$$ (4)

$${VS}_{i}=\frac{e_{i}}{d_{i}}$$ (5)

$${VF}_{i}=\frac{2}{\frac{1}{{VD}_{i}}+\frac{1}{{VS}_{i}}}=\frac{2∙e_{i}}{(n-1)+d_{i}}$$ (6)

where *n* represents the number of vertices included in a network graph and *d*_*i*_ and *e*_*i*_ represent the degrees of each vertex (*V*_*i*_) in the graph (i.e., the total number of intramodular and intermodular links) and the edges of the vertex (i.e., the number of intramodular links), respectively. The three indices for network modules can be used as indices for vertices located on the boundary of a module by resolving these indices for the vertices.

**Fig 1:**
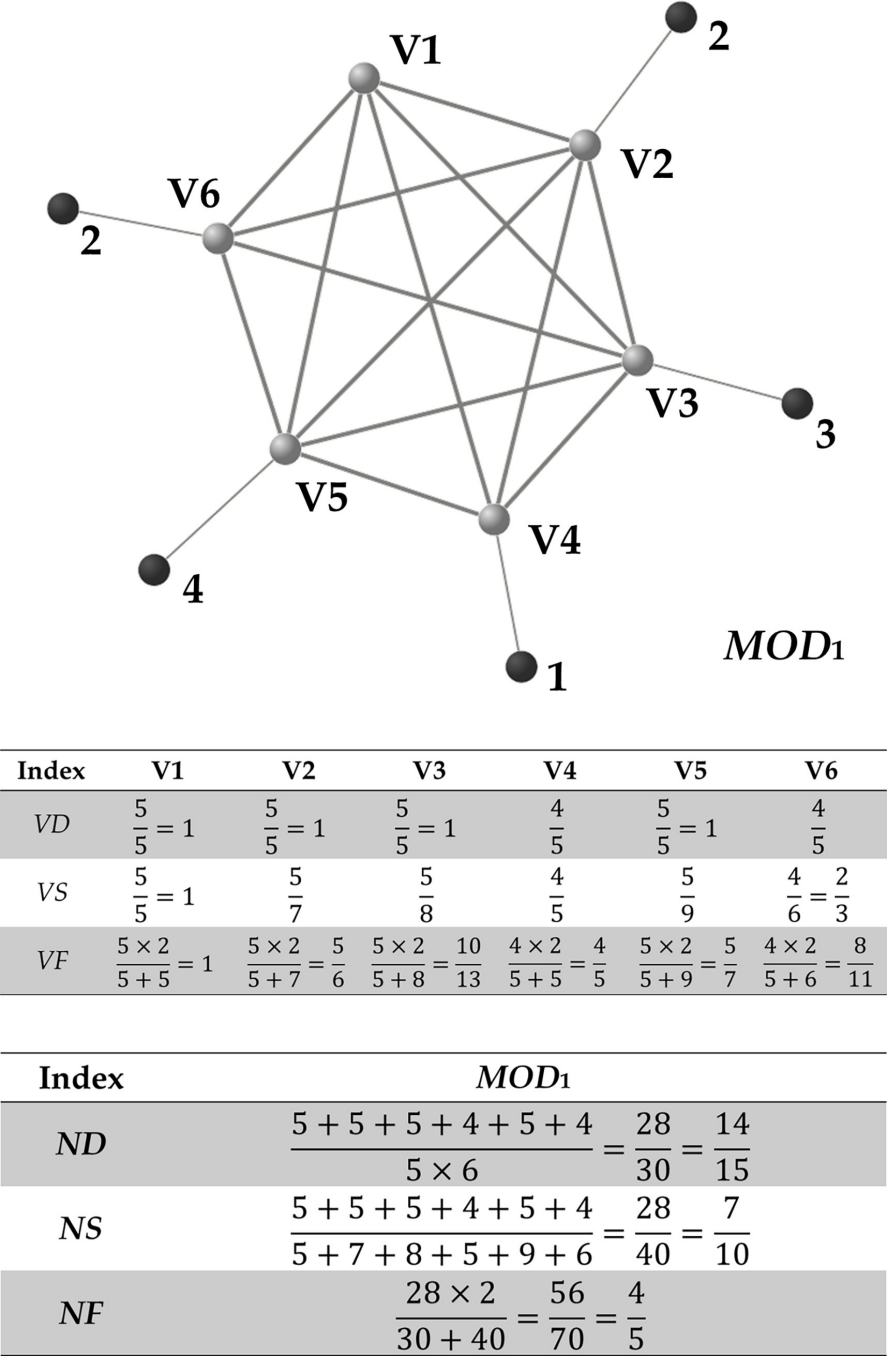
Example calculation of indices using the Confeito algorithm. Lighter vertices represent those included in the network module *MOD*_1_; darker vertices represent those outside of the module; numbers indicate the links from individual vertices included in the module to those outside the module.

To illustrate the roles of these indices in a network graph, we imagined a network module consisting of six vertices (Fig 1). Each vertex in the module (lighter vertices; i.e., V1 to V6) has links to other vertices in the module (intramodular links; darker lines in Fig 1) and links to elements outside the module (intermodular links; lighter lines in Fig 1). When the vertices have intramodular links to all vertices of the module and no intermodular links to other elements, the module is said to be perfect (based on complete intramodular links) and also exclusive (based on the absence of intermodular links). In Fig 1, V4 is not connected to V6, and thus, the network module is not perfect. Also, V2, V4, V5, and V6 have connections to elements outside the module, and thus, the module is not exclusive.

As the network density represents the ratio of intramodular links, it represents the degree of perfection of the network graph. As shown in Fig 1, six elements can be connected by 15 edges and are actually connected by 14 edges; i.e., the network density of the module is 14 over 15.

Network specificity represents the ratio of intramodular links to the total number of links (both intra- and intermodular.), and thus represents the exclusivity of the graph. As shown in Fig 1, the sum of the edges for individual module members is 28 (each edge is counted by two), and the sum of degree for the members is 40 (including 12 links to vertices outside the module: the numbers at the sides of darker nodes represent the number of links from module members to vertices outside of the module). In other words, the network specificity of the module is 28 over 40 (which reduces to 7 over 10).

The network *F*-measure represents the harmonic mean of network density and network specificity, and thus gives an indication of both the degree of perfection and exclusivity. When the network *F*-measure of a network graph is ≥0.5, the graph can be considered a network module based on its degree of perfection and exclusivity. In this case, the average of vertex *F*-measure of the module members is over 0.5, meaning that the majority of links of the average vertex are intra-modular. In conclusion, such a module in total has more tight intra-modular connections compared with intermodular connections. In Fig 1, the network density is 14 over 15 and the network specificity is 7 over 10, and thus the network *F*-measure is 4 over 5 as the calculation shows. This means that the network module is tightly and exclusively connected.

Vertex density, *VD*_*i*_, represents the ratio of the edges of *V*_*i*_, whereas vertex specificity, *VS*_*i*_, represents the ratio of the edges to the degree of *V*_*i*_. Vertex *F*-measure, *VF*_*i*_, represents the harmonic mean of vertex density and specificity, and thus vertex *F*-measure gives an indication of the extent to which the vertex contributes to the network module.

### Initial settings

First, two constants, the expected minimal and maximal sizes of the local communities (or network modules), are set as natural numbers *p* and *q*, respectively. This does not mean that all network module sizes obtained using the Confeito algorithm range from *p* to *q* but that the algorithm can detect a greater number of network modules of sizes ranging from *p* to *q*. This increases the probability of detecting a network module of an appropriate size that contains an element of interest. When a user has no restriction on the sizes of network modules, both values are appropriately set based on topological indices used in ConfeitoGUI. On the other hand, when a user focuses on individual elements in a single network module, the size can be set based on the user’s request. For example, in the field of molecular biology, because such elements are used for experimental validation of the grouping, the size should depend upon the user’s capacity to perform experiments.

### FPO (false-positive-out) series

**Fig 2:**
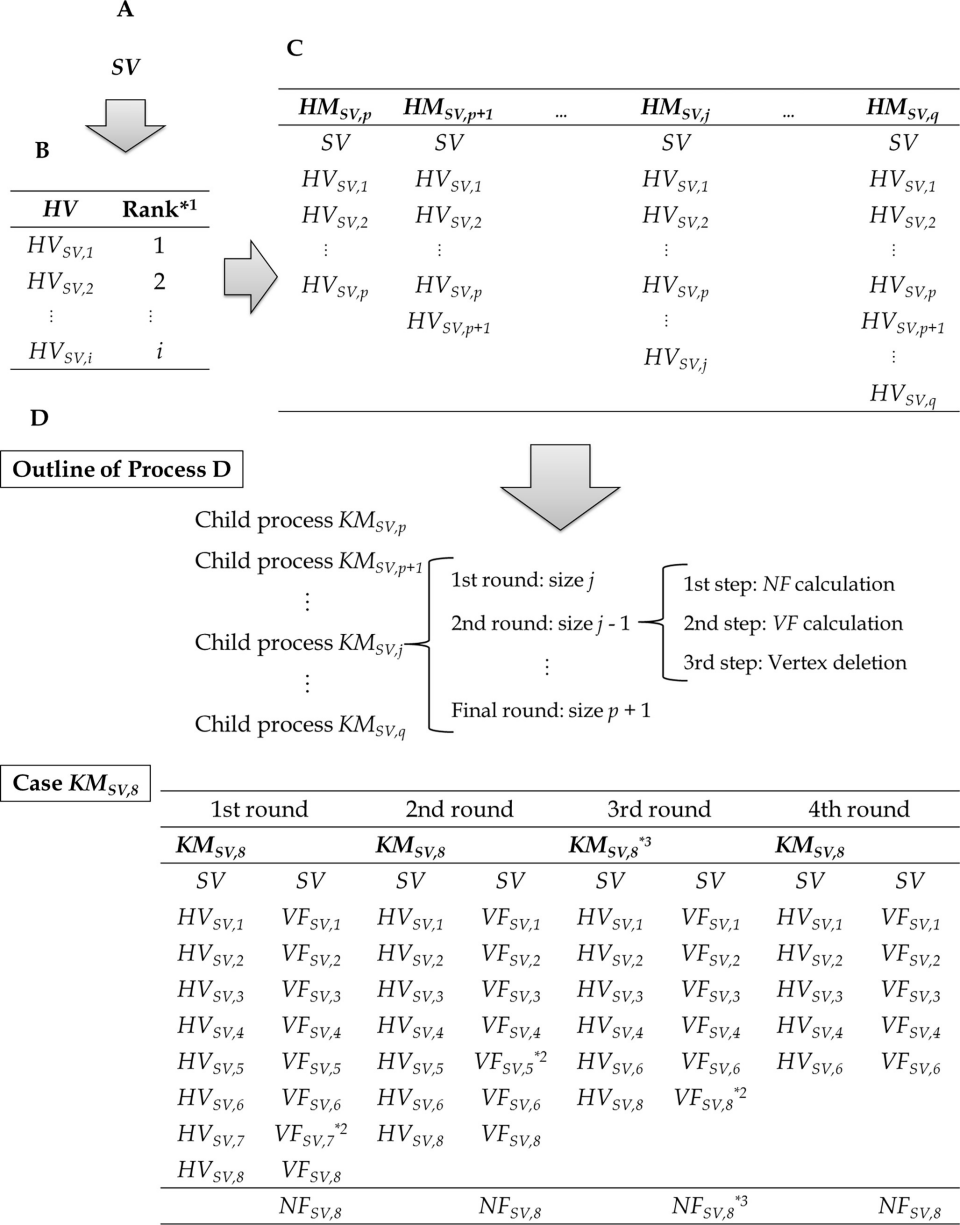
False-positive-out series. **A.** Selection of a seed vertex (*SV*). **B.** Settings of vertices showing high coefficients for correlation with the seed vertex (*HV*s). **C.** Settings of network modules exhibiting high correlations between vertices (*HM*; a highly correlated module). **D.** Identification of the kernel module (*KM*). Outline shows the hierarchy of Process D. The method selects a kernel module (*KM*_*SV*,8_), which originates from a seed vertex and the eight highest vertices (*HM*_*SV*,8_). *^1^ Ranks of coefficients for correlations with an *SV*. *^2^ Lowest *VF* values among those of the vertices in the case of a kernel module (*KM*_*SV*,8_). The vertices are deleted in the next round. *^3^ When the network *F*-measure value (*NF*_*SV*,8_) of the kernel module shows the greatest *NF* value among those calculated throughout the FPO series, the final kernel module (*KM*_*SV*_) and its *NF* value (*NF*_*SV*_) are replaced by *KM*_*SV*,8_ and *NF*_*SV*,8_, respectively.

The FPO series (Fig 2 and S1 Video) identifies vertices that are highly correlated with a given vertex. The process of the series is that first, a single vertex is selected as a seed vertex (process A); second, a vertex group in which members are highly connected to the seed vertex is selected (process B); third, network indices for the group are calculated (process C); fourth, from the group, a vertex with the lowest network index is deleted (process D); and by repeating processes C and D, a final network module originating from the seed vertex is constructed. This series is explained in detail as follows.

A) Selecting a seed vertex (*SV*)
  An arbitrary vertex is selected as an *SV* for the FPO series (Fig 2A).
B) Setting highly correlated vertices (*HV*s)
  A list of correlation coefficients denoting correlations with the seed vertex is selected from a correlation matrix, and the coefficients are aligned in descending order (Fig 2B). The vertices in the list are known as *HV*s, with *HV*_*SV*_,_*i*_ representing a vertex with the *i*th coefficient for correlations with the seed vertex.
C) Setting highly correlated modules (*HM*)
  A group of vertices including the seed vertex and vertices from the first (*HV*_*SV*,*1*_) to the *j*th (*HV*_*SV*,*j*_) is designated a highly correlated module (*HM*_*SV*,*j*_; *p* ≤ *j* ≤ *q*) (Fig 2C). In the module, pairs of vertices that have higher correlation coefficients than that of the seed vertex and the *i*th vertex (*HV*_*SV*,*i*_; 1 ≤ *i* ≤ *j*) pair are connected.
D) Detecting a kernel module (*KM*)
  In this process, a group of vertices that includes the seed vertex and exhibits the maximal (or more precisely, the approximately maximal) *NF* value (known as *NF*_*SV*_) is selected as a final kernel module for the seed vertex (*KM*_*SV*_) (Fig 2D). First, each highly correlated module (*HM*_*SV*,*j*_; *p* ≤ *j* ≤ *q*) is set as the initial kernel candidate module (*KM*_*SV*,*j*_), and a kernel candidate module with the lowest *j* value (*KM*_*SV*,*p*_) at this time is set as the initial kernel module (*KM*_*SV*_). The *NF* value of the temporal kernel module is calculated as the initial *NF*_*SV*_.

For each initial kernel candidate module (*KM*_*SV*,*j*_), a child process for the module is executed. The child process consists of rounds in which a vertex (except for the seed vertex) is repeated until the size of the candidate module is *p* + 1.

Each round consists of the following three steps. 1) The *NF* value of the candidate module (known as *NF*_*SV*,*j*_) is calculated. If the *NF* value is greater than *NF*_*SV*_, *NF*_*SV*_ and *KM*_*SV*_ are replaced with *NF*_*SV*,*j*_ and *KM*_*SV*,*j*_, respectively (see an example for *KM*_*SV*,8_ in Fig 2D). 2) In the candidate module, the *VF* values of all vertices, except for that of *SV*, are calculated (for instance, *VF*_*SV*,5_ is the *VF* value of *HV*_*SV*,5_). 3) The vertex representing the minimal *VF* value is deleted from the candidate module. In other words, the size of the candidate module decreases by 1 in this step. If the size is greater than *p* + 1, the first step is executed again.

Finally, the kernel module (*KM*_*SV*_) is selected as the best network module originating from the seed vertex, and the *VF* values of the vertices (*HV*_*SV*,*i*_) included in the kernel module are calculated as *VF*_*SV*,*i*_.

### Merging kernel modules (modularizing series)

**Fig 3:**
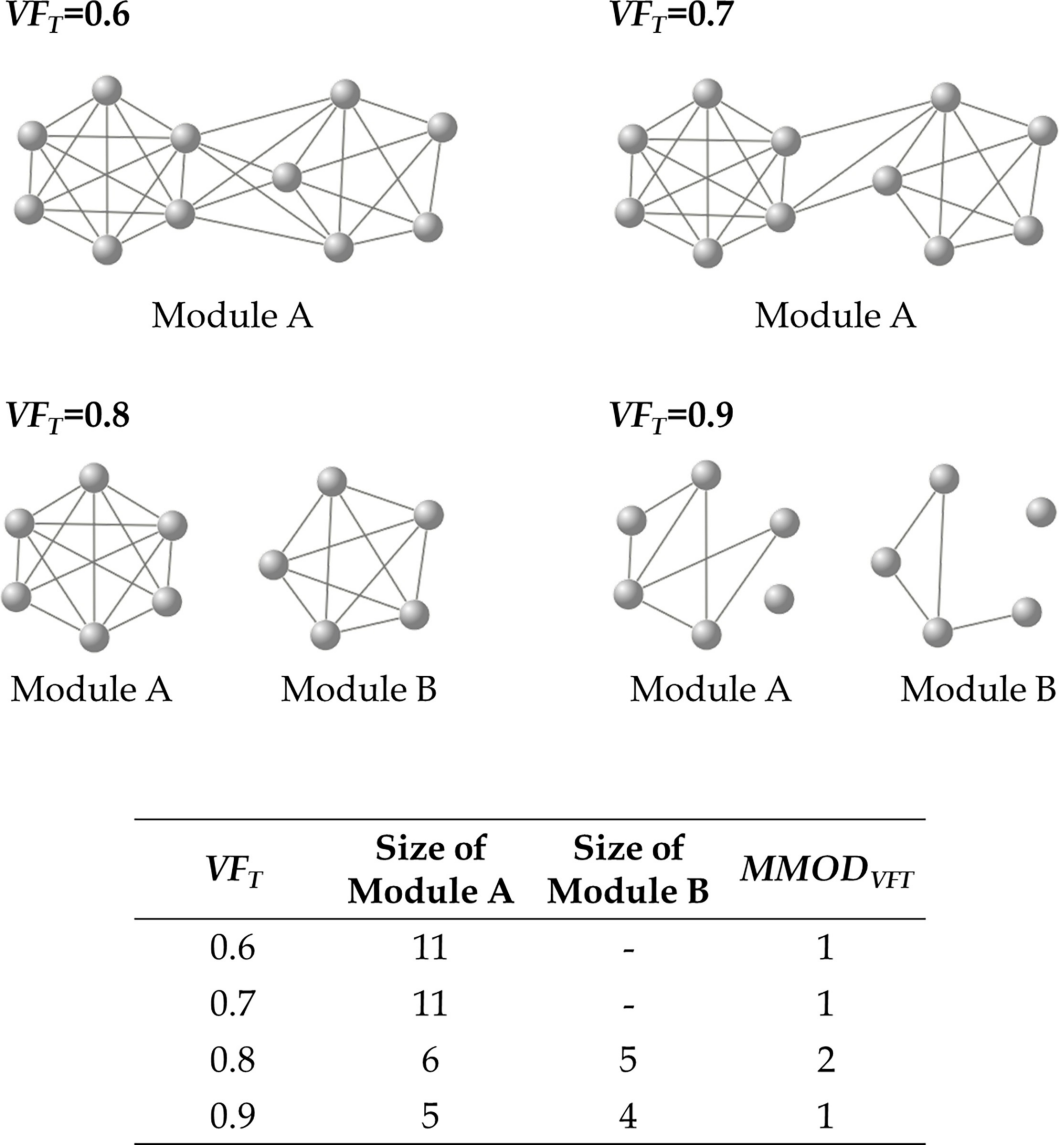
Example of a modularizing series. *VF*_*T*_ ranges between 0.6 and 0.9 in steps of 0.1. Values of *p* and *q* are set to 5 and 50, respectively. At *VF*_*T*_ values of 0.6 and 0.7, there is a single network module, module A. At *VF*_*T*_ values of 0.8 and 0.9, the single network module separates into two network modules, A and B. The sizes of both network modules at the former *VF*_*T*_ value are greater than *p*, indicating that both network modules are valid. However, the size of network module B at the latter *VF*_*T*_ value is smaller than *p*, indicating that at *VF*_*T*_ = 0.9; therefore, only module A is valid. This indicates that the network modules at *VF*_*T*_ = 0.8 should be set as the final network graph (*MOD*) in this example.

Kernel modules (*KM*_*SV*_s) originating from multiple *SV*s show redundancy in terms of the memberships of the modules. To eliminate this redundancy, the kernel modules are merged according to the following processes (Fig 3).

A) Depicting a network graph with a specific *VF* threshold
  For merging kernel modules, a new network graph is plotted based on a specific *VF* (*VF*_*T*_) threshold instead of the correlation coefficients among these vertices. When the value of *VF*_*SV*,*i*_ is equal to or greater than *VF*_*T*_, edges between a seed vertex and the *i*th vertex for the seed vertex (*HV*_*SV*,*i*_) are connected. Specifically, in a kernel module originating from the seed vertex, only edges connected to the seed vertex are used for this process. Through the process, vertices shared with multiple kernel modules are united. Thus, the redundancy of vertices in kernel modules is eliminated in the network graph depicted in the process.
B) Depicting network graphs with various thresholds of *VF*
  Network graphs with various *VF*_*T*_ thresholds (*MOD*_*VFT*_) are created. For instance, for *VF*_*T*_ ranging from 0.50 to 0.99 in steps of 0.01, a total of 50 network graphs of *MOD*_*VFT*_ with different thresholds are created.
C) Selecting the best modules
  In *MOD*_*VFT*_, network modules with sizes ranging from *p* to *q* are those initially desired and the number of the modules (*MMOD*_*VFT*_) is counted. *MOD*_*VFT*_ showing the maximal number is designated as the final network module (*MOD*). The number of network modules included in *MOD* is set to *MMOD*.

Although the present algorithm constructs network modules using *VF* values as indices to connect vertices instead of correlation coefficients, the *NF*s of *MOD* and the *VF*s of the vertices included in *MOD* are calculated using the original correlation coefficients. That is, the network modules included in *MOD* are represented using vertex–vertex connections based on such coefficients.

### FNI (false-negative-in) series

**Fig 4:**
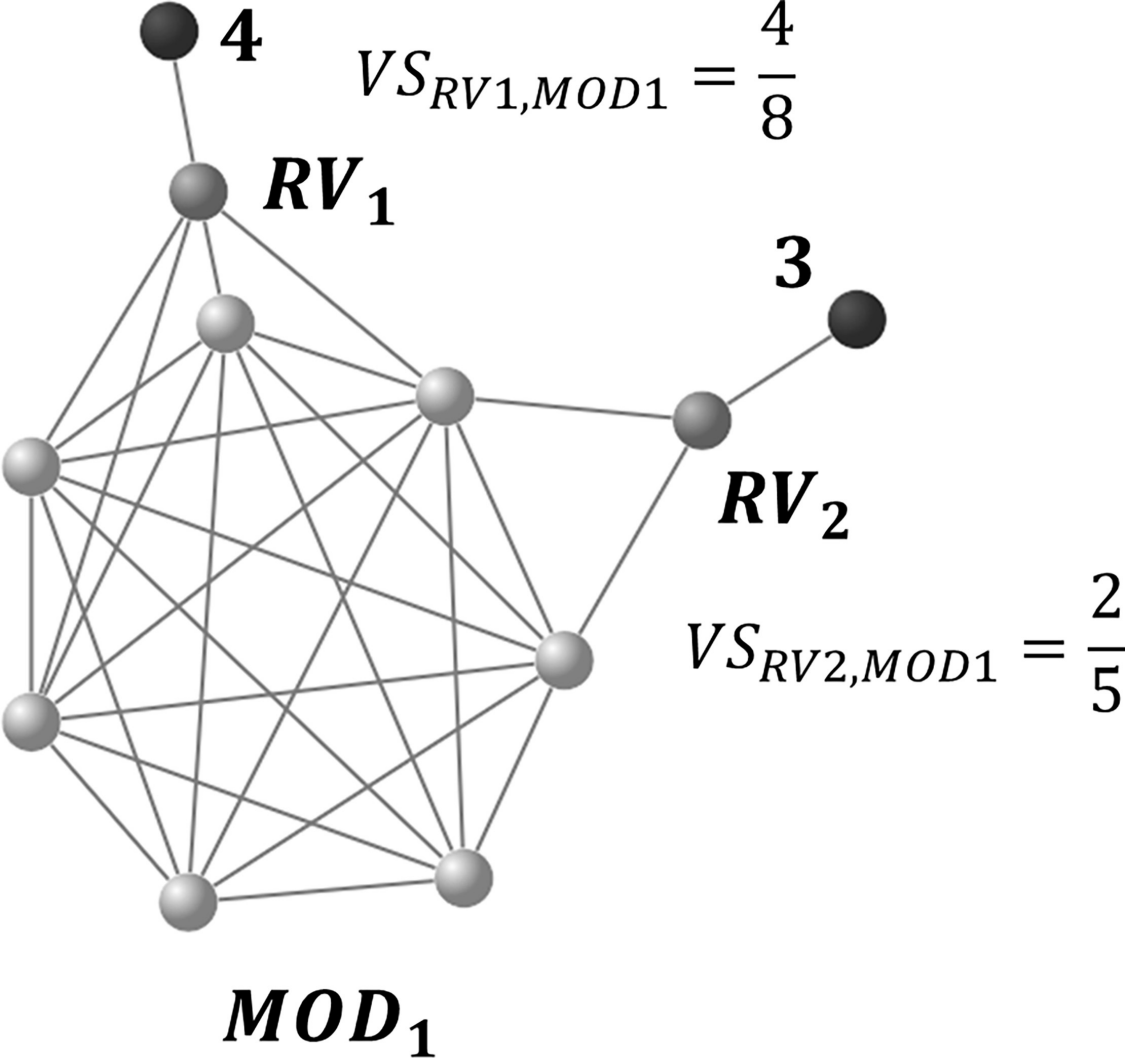
Example of an FNI series. Lighter vertices represent those included in *MOD*_1_. Darker vertices, *RV*_1_ and *RV*_2_, represent candidate false-negative vertices. Black vertices represent a group of vertices with connections to the candidates, in which numbers associated with the groups represent the number of vertices included in the respective group. Fractions with darker vertices represent *VS* values (*VS*_*RV*1,*MOD*1_ and *VS*_*RV*2,*MOD*1_). At a *VS* threshold of 0.5, only *RV*_*1*_ is incorporated into *MOD*_*1*_ as a false-negative vertex.

It is possible to obtain false-negative indices that are closely related to a network module but not included through the FPO and the modularizing series. In the FNI series (Fig 4), such vertices are detected for each network module.

Each network module included in *MOD* is set as *MOD*_*i*_; where 1 ≤ *i* ≤ *M*_*MOD*_. Vertices that are not included in *MOD*_*i*_ are set to residual vertices (*RV*_*j*_; 1 ≤ *j* ≤ *N*–*N*_*MODi*_, and *N* and *N*_*MODi*_ represent the number of vertices included in the whole network and *MOD*_*i*_, respectively). The *VS* value of a residual vertex to the network modules in *MOD* is calculated in descending order of highly correlated vertices for the residual vertex (*HV*_*RVj*_). The last highly correlated vertex to be used for the calculation is preliminarily determined: in the ConfeitoGUI toolkit, a user can set the number of vertices (the default value is 1000). Although *VS* values of the residual vertex should be calculated for all network modules in *MOD*, it is time-consuming to execute such calculations for each network module. The toolkit provides a result equivalent to the calculation for all network modules by repeated calculation for only the network module including the next highly correlated vertex for the residual vertex. The process requires a series of calculations only for a single set of highly correlated vertices for the residual vertex. The vertex with a VS value to a network module greater than an arbitrary value (e.g., 0.5) is incorporated into the module as a “false-negative” vertex.

The FNI series is not only useful for identifying false-negative elements, but also for analyzing relationships between heterogeneous elements. For example, the FNI series can be used to identify network modules of homogeneous elements using FPO and then identifying heterogeneous elements (or elements that are not included among the homogeneous elements) that are specifically related to network modules.

## Instructions for use of ConfeitoGUI

The present version of the ConfeitoGUI package (1.2.0 as of September 2017) requires Windows 7 Professional (64-bit) and an Intel Core2 Quad CPU Q9400 @ 2.66 GHz for Windows or OS X Yosemite 10.10.3 and Intel Core i7 @ 2.2 GHz for Macintosh. Both operating systems require at least 8 GB of memory and Java 8 Update 45 (64-bit). The package is available at http://www.plant.osakafu-u.ac.jp/~kagiana/confeito/. The present version of ConfeitoGUI is not designed for the Linux operating system.

### For quick use

The quick use method for ConfeitoGUI is illustrated on our website (http://www.plant.osakafu-u.ac.jp/~kagiana/confeito/test.html). The website provides a method to use it with its snapshots and a test dataset.

### Input

ConfeitoGUI accepts multivariate datasets (i.e., two-dimensional tables containing elements as rows and datasets as columns). Although the Confeito algorithm accepts any type of correlation coefficient, ConfeitoGUI is equipped with a “Correlation Tool” that enables calculation of both Pearson and cosine correlation coefficients.

### The following rapid steps can be followed to use ConfeitoGUI:

1. ConfeitoGUI installation and uninstallation
  From the website (http://www.plant.osakafu-u.ac.jp/~kagiana/confeito/ and http://webs2.kazusa.or.jp/kagiana/confeito/), download ConfeitoGUI_1.2.0.zip and unzip it to a local drive.
2. Java Runtime Environment (JRE) installation
  Install a new 64-bit version of JRE. As of September 2017, the software is available at http://www.oracle.com/technetwork/java/javase/downloads/index.html.
3. Execution
  Double click the “launch.bat” or “launch_mac.command” file for Windows or Macintosh, respectively, to open the “ConfeitoGUI” window.
4. Calculate a correlation matrix
  Click the “Init Tool” menu tab and then click “Correlation Tool” to begin calculation of a correlation matrix. In the “Correlation Tool” window, browse for an input file (e.g., a sample dataset available at the ConfeitoGUI website) by clicking “Browse” in the “Input File” row. Next, select an output folder by clicking “Browse” in the “Output Folder” row. Finally, click “Start” to calculate a correlation matrix in a new folder based on the input file.
5. Preprocessing
  Click “Preprocessing Tool” to create a text-formatted file including information on file paths of the correlation dataset. Click “Browse” in the “Correlation Data Folder” row to select the folder created in step 4. Next, click “Browse” to create an intermediate file (*.flis). Finally, click “Start.”
6. False-positive-out (FPO) analysis
  Click the “Confeito Analysis” tab. Select “FPO” in the “Analysis Menu,” click “Browse” in the “Correlation Data File” row to open the intermediate file created in step 5, and then click “Browse” in the “Output Folder” row to select an output folder. Finally, click “Start” to create several FPO analysis results files.

### Output

One of the output files will be named “result.net,” and this file can be used to depict a network constructed by ConfeitoGUI using a network drawing software package such as Pajek [18]. The result.net file is formatted for Pajek and includes information regarding vertices and edges between vertices. The user can arrange the positions of vertices using Pajek.

Another output file will be named “result.mod,” and this file includes information regarding all network modules constructed by ConfeitoGUI. In the result.mod file, the *NF* values (i.e., the harmonic mean of *ND* and *NS*) of network modules and the *VF* values (i.e., the harmonic mean of *VD* and *VS*) of the vertices in the network modules (calculated using Process C in the modularizing series) are indicated. The higher the *NF* value of a network module, the tighter the intramodular connections will be in the module. A vertex with a higher *VF* represents a more central element in the module.

## Results

We compared ConfeitoGUI’s accuracy to that of other local community identification methods (e.g., the Louvain [4], simulating annealing [3,17], and fast greedy [18] methods) using network modules from a large mouse microarray dataset including results from 37,013 Affymetrix mouse microarray samples (named GPL1261) that was obtained from the Gene Expression Omnibus (GEO) of NCBI in April 2014 (S1 Table). Cosine correlation coefficients between experiments were calculated, and a correlation network was constructed using the coefficient matrix. The microarray samples’ network modules were detected using FPO analysis with parameters of 6 for the minimum and 100 for the maximum elements. In addition, network modules were identified using default setting of the Louvain method with Pajek software [23] and the simulating annealing and fast greedy methods with R software. The resulting network modules were compared through a series of microarray samples using the *F*-measure for their memberships, because in this comparison similarity in sizes between original experimental groups and expected local communities is thought to be true. The *F*-measure index is the harmonic mean of the precision and recall indices [24]. The precision index was calculated as the proportion of samples shared between a network module and a single series of samples to the number of samples in the module. The recall index was calculated as the ratio of shared samples to the number of samples included in the series. High *F*-measure values are indicative of a high degree of similarity between a network module and a sample series, which provides greater accuracy in discriminating between closely related experiments. The *F*-measure was calculated for all network modules obtained using all methods. The network modules obtained using ConfeitoGUI exhibited greater average *F*-measure values than those obtained using the other methods (Table 1), suggesting that ConfeitoGUI performs better than the other publicly available tools in terms of identifying network modules with sizes more appropriate to the original experimental groups.

**Table 1:** Comparison of the ConfeitoGUI to other tools used for size-sensitive module identification.

Method/Tool	Correlation coefficient*	*F*-measure**
ConfeitoGUI	–	0.605 ± 0.007
Louvain[12]	0.97	0.465 ± 0.008
Simulating annealing[13,14]	0.96	0.397 ± 0.008
Fast greedy[15]	0.96	0.391 ± 0.008

* Correlation coefficients represent thresholds for identifying networks for correlations in mouse microarray samples using methods other than the ConfeitoGUI tool, as the ConfeitoGUI tool uses the index modified with the correlation coefficient.

** For the average and standard error of the *F*-measure values, *n* = 2274.

**Fig 5:**
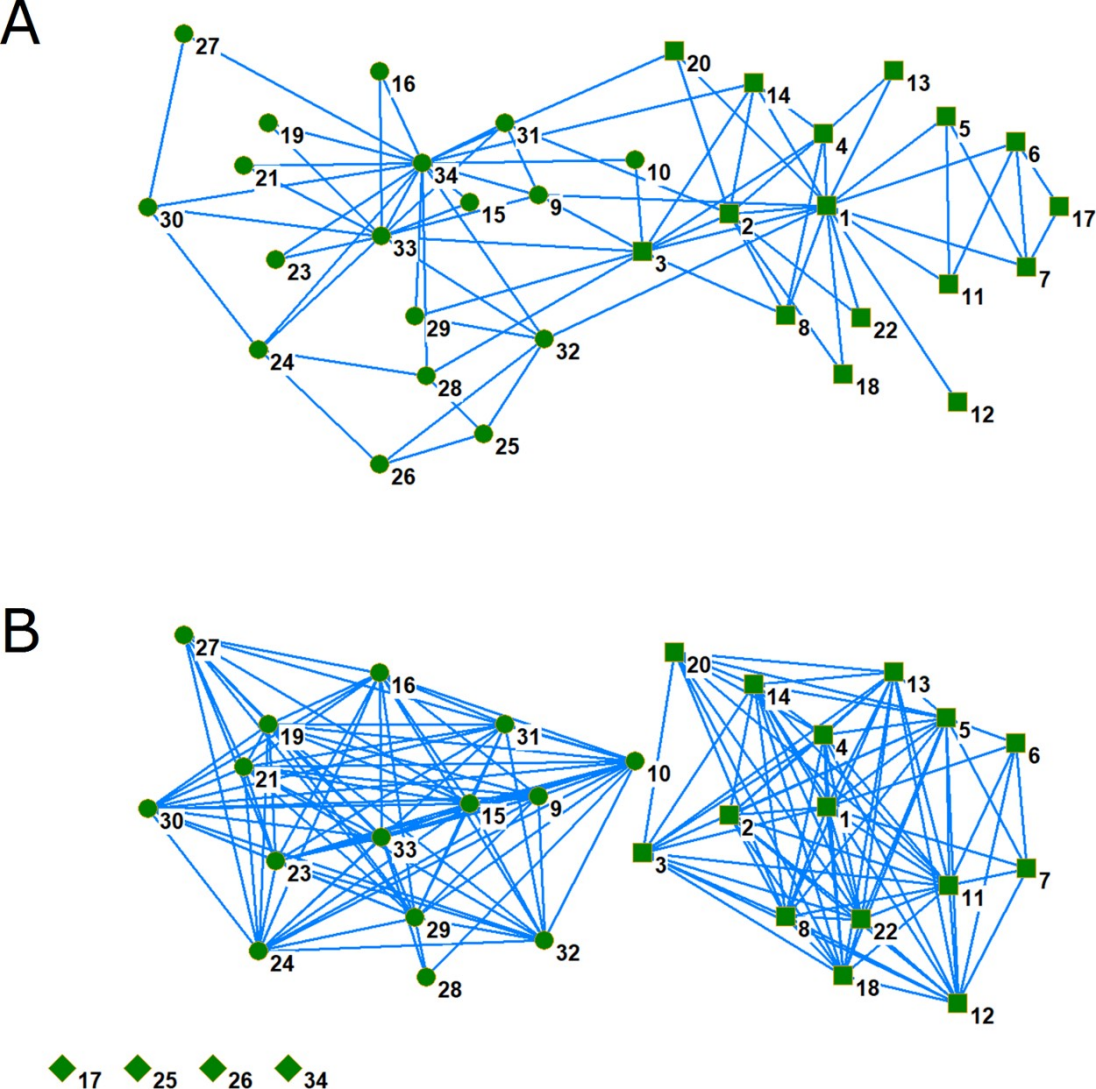
Application to a dataset of Zachary’s karate club. (A) Original relationships between the karate club’s members. Circle and square vertices represent a group in which members numbered 33 and 34 are located in the center and another group in which members numbered 1 and 2 are located in the center, respectively. (B) The result of the FPO series, in which 30 members were successfully separated into two groups and 4 members (diamonds) were unclassified. These unclassified members were successfully assigned through the following FNI series. The coordinates of members are common with both network graphs, except for those of the four residual members.

ConfeitoGUI is applicable not only to datasets in the field of molecular biology but to those in social networks. We applied the toolkit to the dataset of Zachary’s karate club provided by Newman et al. [3] to compare it with their result (Fig 5; see also our website for details, http://www.plant.osakafu-u.ac.jp/~kagiana/confeito/test4.html). Although they provided a result in which one member of the club (No. 10) was misclassified, their following report [5] showed accurate classification. Using parameters of 2 for the minimum (*p*) and 30 for the maximum vertices (*q*) and Pearson correlation to apply ConfeitoGUI to the dataset, the toolkit provided the correct classification (Fig 5B for the FPO series and Table 2 for the FNI process). Both values of *p* and *q* were set as wide as possible in the range of the number of members. In the FPO series, 30 members were successfully separated into two groups and 4 members were unclassified because of their relationships to the members classified into the two groups. Although the member numbered 34 is a central member in one separated group, the member’s friendship is dissimilar to those of other members; i.e., the member is related to more members than the others in the group. The FNI series led to the exact classification of the residual members by calculating their *VS* values for the two groups. ConfeitoGUI is useful for border-sensitive community detection by selecting elements on boundaries and classifying them based on their *VS* values for network modules.

**Table 2:** The FNI series to a dataset of Zachary’s karate club.

Residual member*	Targeted member**	*VS*
17	5	1.0
25	24	1.0
26	28	1.0
34	33	1.0

* represents 4 residual members after the FPO series to the dataset.

** represents a member with the highest correlation coefficient for a residual member.

## Discussion

The ConfeitoGUI toolkit described here is a powerful tool for creating many local communities in which the size is based on user requests from a network. The toolkit accepts any type of multivariate data. By changing simple parameters that represent sizes of interest for the toolkit, users can adjust the sizes of local communities that include any element of interest. When changing the maximum size for a network module (*q*) into a smaller number, network modules with their sizes larger than the number can be divided into smaller network modules. On the other hand, when changing *q* into a larger number, multiple network modules can be merged to create a larger network module. ConfeitoGUI performed better than other publicly available tools in terms of identifying network modules and their appropriate sizes using indices based on the improved Confeito algorithm as shown in Table 1.

Although the algorithm was developed to identify local communities from within a large network, the present version of ConfeitoGUI can also be used to classify all of the elements within a large network by adding a modularizing series. In the first series (i.e., the FPO series), local communities originating from individual elements are obtained regardless of those originating from other elements. Therefore, the elements included in these communities are redundant with respect to each other. In the second series (i.e., the modularizing series), this redundancy is eliminated by merging the local communities. Through implementation of these series, elements exhibiting relatively poor correlation with the members of the merged communities are eliminated, even if they show high affinity for the community members. In the third series (i.e., the false-negative-in [FNI] series), elements among those not included in any of the communities but that exhibit high affinity for the merged communities are incorporated into the communities. By executing these series, all elements in a network can be classified into local communities.

## Extensions and challenges

ConfeitoGUI can accept heterogeneous (or different types of) elements (e.g., genes and metabolites) by combining FPO and FNI analyses. In general, when heterogeneous elements are used for a network analysis, network modules tend to be composed of either type of element (i.e., there are almost all network modules containing only genes or those containing only metabolites). To analyze relationships between different types of elements as well as within a type, the following steps are recommended for dealing with heterogeneous elements. First, a network graph consisting of homogeneous (or a single type of) elements (e.g., genes) is plotted using the results of the FPO analysis. Heterogeneous (or another type of) elements (e.g., metabolites) are then mapped onto the network using the results of the FNI analysis (e.g., based on gene-metabolite correlations). Next, correlation matrices representing gene-gene and gene-metabolite relationships are created using the “Correlation Tool” in ConfeitoGUI. Moreover, a gene-gene correlation matrix is used for FPO analyses of genes, whereas a gene-metabolite correlation matrix is used for FNI analyses to detect gene-metabolite relationships. However, analyses of relationships between heterogeneous elements using ConfeitoGUI are complex. A function that will enable simple accommodation of such heterogeneous elements will be added to the ConfeitoGUI toolkit in the future.

It can be challenging to identify local communities from within large datasets using the ConfeitoGUI toolkit. The software initially generates a correlation matrix for all elements. When the number of elements is quite large (e.g., currently more than 100,000), it is difficult to create a correlation matrix and execute the software analysis within a reasonable time frame when using a personal computer (PC). For instance, in the case of a typical genome, the number of genes is lower than this limit (i.e., <100,000). The number of elements that can be accepted by the software is limited by the memory in the user’s PC. A useful approach to overcome this limitation is to preliminarily classify the elements, for instance according to topology, as is the case with the Louvain method.

The present version of ConfeitoGUI is equipped with the FNI series only for a single network module. To classify all elements in a network into a network module, the next version of the toolkit will provide a function for such classification.

## Supporting information

S1 Video Steps of the false-positive-out series. (MP4)

S1 Table A microarray dataset for comparative analysis. The manual of ConfeitoGUI in English is available at our site (http://www.plant.osakafu-u.ac.jp/~kagiana/confeito/).
(XLSX)
